# Variation in the Maternal Corticotrophin Releasing Hormone-Binding Protein (CRH-BP) Gene and Birth Weight in Blacks, Hispanics and Whites

**DOI:** 10.1371/journal.pone.0043931

**Published:** 2012-09-11

**Authors:** Pathik D. Wadhwa, Hyagriv N. Simhan, Sonja Entringer, Claudia Buss, Roger Smith, Calvin J. Hobel, Naveed Farhana, Lawrence Shimmin, James E. Hixson, Charles F. Sing

**Affiliations:** 1 Departments of Psychiatry and Human Behavior, University of California Irvine, Irvine, California, United States of America; 2 Department of Pediatrics, University of California Irvine, Irvine, California, United States of America; 3 Department of Obstetrics and Gynecology, University of California Irvine, Irvine, California, United States of America; 4 Department of Epidemiology, University of California Irvine, Irvine, California, United States of America; 5 Department of Obstetrics, Gynecology and Reproductive Sciences, University of Pittsburgh, Pittsburgh, Pennsylvania, United States of America; 6 Mothers and Babies Research Centre/Endocrine Unit, The University of Newcastle, Newcastle, New South Wales, Australia; 7 Department of Obstetrics, Gynecology and Pediatrics, Cedars Sinai Medical Center and the David Geffen School of Medicine, University of California Los Angeles, Los Angeles, California, United States of America; 8 Department of Epidemiology, Human Genetics and Environmental Sciences, The University of Texas Health Science Center, Houston, Texas, United States of America; 9 Department of Human Genetics, University of Michigan, Ann Arbor, Michigan, United States of America; Institute of Zoology, Chinese Academy of Sciences, China

## Abstract

**Background:**

Given the unique role of the corticotrophin-releasing hormone (CRH) system in human fetal development, the aim of our study was to estimate the association of birth weight with DNA sequence variation in three maternal genes involved in regulating CRH production, bioavailability and action: CRH, CRH-Binding Protein (CRH-BP), and CRH type 1 receptor (CRH-R1), respectively, in three racial groups (African-Americans, Hispanics, and non-Hispanic Whites).

**Methods:**

Our study was carried out on a population-based sample of 575 mother–child dyads. We resequenced the three genes in mouse–human hybrid somatic cell lines and selected SNPs for genotyping.

**Results:**

A significant association was observed in each race between birth weight and maternal CRH-BP SNP genotypes. Estimates of linkage disequilibrium and haplotypes established three common haplotypes marked by the rs1053989 SNP in all three races. This SNP predicted significant birth weight variation after adjustment for gestational age, maternal BMI, parity, and smoking. African American and Hispanic mothers carrying the A allele had infants whose birth weight was on average 254 and 302 grams, respectively, less than infants having C/C mothers. Non-Hispanic White mothers homozygous for the A allele had infants who were on average 148 grams less than those infants having A/C and C/C mothers.

**Conclusions:**

The magnitudes of the estimates of the birth weight effects are comparable to the combined effects of multiple SNPs reported in a recent meta-analysis of 6 GWAS studies and is quantitatively larger than that associated with maternal cigarette smoking. This effect was persistent across subpopulations that vary with respect to ancestry and environment.

## Introduction

The contribution of genetic and environmental determinants to variation in birth weight is an area of considerable on-going interest and investigation. The association of pathophysiological fetal growth, as reflected by extremes of the birth weight distribution (small-for-gestational age (SGA) and large-for-gestational age (LGA) births), with high perinatal morbidity and mortality has long been established [1]. More recent studies suggest that variation in the normal range of variation in fetal growth (birth weight assessed as a quantitative trait) is also associated with many important developmental and health outcomes (e.g., childhood and adult blood pressure, body composition and metabolic function [2], [3]).

While much effort has been committed to the identification and management of pathologically inadequate or pathologically excessive fetal growth, less effort has been committed to evaluating the determinants of normal variation in fetal growth and birth weight. Furthermore, current limitations in knowledge about factors influencing variation in fetal growth make it difficult to accurately distinguish the optimally grown from the sub- or supra-optimally grown infant [4].

Birth weight, the phenotype representing the culmination of fetal growth, is a complex, multi-factorial trait regulated by the interplay of maternal and fetal genes and intrauterine physiology (endocrine, immune, vascular and other processes). Relatively little information, however, is currently available about the genetic loci that explain variation in birth weight. Studies using animal models have provided important insights regarding the genetic and environmental determinants of birth weight including the role of imprinted genes [5]. However, a significant limitation in the ability to generalize findings from animal models (even among closely-related mammals) to humans is a consequence of the observed large inter-species variation in the physiology of pregnancy [6]. One important example of this inter-species variation is exemplified by the corticotrophin-releasing hormone (CRH) family of proteins.

CRH is the key regulator of the hypothalamic-pituitary-adrenal axis. It is primarily secreted centrally (in the brain) and exerts major effects on growth, reproduction, immunity, thyroid function and metabolism [7], [8]. In the context of pregnancy in primates, but not other mammals, the placenta is a major peripheral site of CRH production. Placental CRH is released into the maternal as well as fetal compartments, where it exerts its biological actions. However, even across primates (e.g., between New and Old World monkeys and humans), there are large differences in the patterns of production, activity and regulation of placental CRH and CRH binding protein [6], [9], [10].

In human pregnancy CRH is primarily produced in the decidua, fetal membranes and placenta [11]. CRH production increases in an exponential manner over the course of human gestation, and it is released into both maternal and fetal compartments. A 37-kDa CRH binding protein (CRH-BP) is produced in all mammals. The primary peripheral source of human CRH-BP production during pregnancy is the maternal liver. CRH-BP binds CRH with equal or greater affinity than the CRH receptors, resulting in dimerization of the protein and clearance of CRH from the circulation and inhibiting its function [12], [13], [14]. This places CRH-BP in an important regulatory position between CRH and its receptors [15]. Maternal CRH-BP levels do not change significantly during most of gestation, but they fall during the final weeks of normal pregnancy [16], [17]. CRH exerts its biological effects by activating two G-protein-coupled receptors: CRH receptor 1 (CRH-R1), primarily expressed in the myometrium and fetal membranes, and CRH receptor 2 (CRH-R2), which has a higher affinity for urocortin and appears to play a role in vascular control [18], [19]. Thus, the genes encoding CRH, CRH-BP and CRH-R1 represent the key genetic regions regulating CRH production, bioavailability and activity in the context of human gestation.

In humans, placental CRH is known to play an important role in outcomes related to the length of human gestation and timing of onset of parturition [6], [20]. Evidence suggests CRH may also play a role in fetal growth and birth weight via direct as well as indirect pathways. We have previously reported that inter-individual variation in placental CRH production in mid-gestation predicts variation in birth weight [20]. Other work has demonstrated cross-sectional associations between variation in birth weight and cord levels of CRH at birth [21]. Yet other studies have demonstrated relationships between placental CRH and risk for obstetric conditions, such as preeclampsia and pregnancy-induced hypertension [22], [23], [24], that, in turn, are predictive of extreme birth weight. A recent study convincingly demonstrated that CRH, acting via the CRH-R1 receptor, stimulates trophoblast production of GLUT1 - the rate-limiting determinant of placental glucose transport – thereby supporting its direct role in fetal growth [25], [26]. Thus, these findings, coupled with findings relating CRH to other endocrine, immune and vascular processes that are known to play a role in regulating processes underlying fetal growth, provide the rationale and biological plausibility for the study reported here.

Given the importance of elucidating the determinants of birth weight and the potentially unique role of the CRH system in human fetal growth and development, we carried out the study reported here to estimate the association of variation in birth weight with DNA sequence variation in gene regions coding for the three key genes regulating CRH production, bioavailability and action (i.e., CRH, CRH-BP and the CRH type 1 receptor (CRH-R1), respectively [8], [27]. Our study involved a population-based cohort of mother-child pairs sampled from the three major racial/ethnic populations in the United States (African Americans, henceforth denoted Blacks, Hispanics and non-Hispanic Whites, henceforth denoted Whites).

There are significant differences among racial/ethnic groups for average birth weight [28]. Although we expect the same genetic loci and environmental agents to be involved in determining fetal growth in all pregnancies, the observed average racial/ethnic differences in birth weight may be explained by racial/ethnic differences in the relative frequencies and/or sizes of the effects of variations in these factors. For these reasons, the relative contribution of variation in genetic and environmental factors to variation in birth weight within racial/ethnic cannot be taken as an explanation for differences in average birth weight between racial/ethnic groups. Hence, our goal here is to identify one or more genetic variations that explain birth weight variation in all three racial/ethnic groups.

Because an important goal of clinical research is to identify measures of risk before, or during pregnancy, we elected here to investigate the utility of the maternal genotype in explaining variation in birth weight. We treated birth weight as a quantitative trait in order to evaluate the determinants of normal, physiological fetal growth. In addition to the dominant effect of gestational age at birth, the pursuit of defining the agents that influence fetal growth has identified a significant contribution of a subset of predictors of birth weight that include sex of the child and maternal BMI, race/ethnicity, parity, obstetric risk and smoking [29]. We therefore included these risk factors in our analyses of the impact of genetic variation on explaining variation in birth weight. We addressed the following research questions:

1. Does the CRH pathway-related genetic information explain variation in birth weight after accounting for the effects of other established risk factors/determinants of birth weight?
2. Is there CRH-pathway related genetic information that explains variation in birth weight across the three major racial/ethnic groups in the U.S.?

## Materials and Methods

### Study Participants

Participants in our study were recruited at two study sites in Southern California (the University of California, Irvine, Medical Center in Orange, CA, and the Cedars Sinai Medical Center in Los Angeles, CA) and one study site in Pennsylvania (the Magee Womens Hospital in Pittsburgh, PA). All participants in the current study participated in one or more clinical studies of pregnancy outcomes. These study protocols included multiple, serial assessments of biomedical, biological, psychosocial, and behavioral processes in pregnancy. Women <18 years, with multiple gestation, cord, placental, or uterine anomalies, fetal congenital malformations and those whose pregnancies ended in a spontaneous abortion were ineligible for the study. Beyond study design, we also restricted the final study dataset to only include Blacks, Hispanics, and Whites; and removed births <168 days (24 weeks) gestation. Race/ethnicity was determined by self-report using the Office of Management and Budget 1997 Revisions to the Standards for the Classification of Federal Data on Race and Ethnicity (Revision of Statistical Policy Directive No. 15, Race and Ethnic Standards for Federal Statistics and Administrative Reporting). The characteristics of the samples ascertained for each racial group are presented in Table 1. Our study is based on the analyses of 575 unique mother-child pairs.

**Table 1:** Subject characteristics.

	Blacks	Hispanics	Whites	Pooled
Mother/child pairs available	188	144	243	575
Recruited from Irvine, CA	54 (28.7)	128 (88.9)	158 (65.0)	340 (59.1)
Recruited from Pittsburgh, PA	134 (71.3)	16 (11.1)	85 (35.0)	235 (40.9)
**Maternal Characteristics**				
Age (years)				
Average (SD)***	26.9 (5.2)	27.6 (5.6)	29.6 (5.8)	28.2 (5.7)
<25 (N, %)***	79 (42.0)	48 (33.3)	64 (26.3)	191 (33.2)
25–33 (N, %)	81 (43.1)	66 (45.8)	109 (44.9)	256 (44.5)
>33 (N, %)	22 (11.7)	26 (18.1)	67 (27.6)	115 (20.0)
Missing	6 (3.2)	4 (2.8)	3 (1.2)	13 (2.3)
Height (cm)				
Average (SD)***	163.6 (6.8)	160.4 (7.3)	165.0 (6.9)	163.4 (7.2)
Pre-pregnancy Weight (kg)				
Average (SD)**	74.7 (18.9)	71.5 (18.3)	69.0 (16.7)	71.4 (18.0)
Pre-pregnancy BMI (kg/m^2^)				
Average (SD)***	27.8 (6.6)	27.8 (6.9)	25.3 (5.8)	26.8 (6.5)
≤19.8 (N, %)**	12 (6.4)	11 (7.6)	29 (11.9)	52 (9.1)
19.8 to 26.0 (N, %)	78 (41.5)	56 (38.9)	127 (52.3)	261 (45.4)
26.1 to 29.0 (N, %)	20 (10.6)	21 (14.6)	32 (13.2)	73 (12.7)
>29.0 (N, %)	70 (37.2)	52 (36.1)	53 (21.8)	175 (30.4)
Missing	8 (4.3)	4 (2.8)	2 (0.8)	14 (2.4)
Parity (N, %)***				
Nulliparous	37 (19.7)	46 (31.9)	109 (44.9)	192 (33.4)
Multiparous	146 (77.7)	94 (65.3)	134 (55.1)	374 (65.0)
Missing	5 (2.6)	4 (2.8)	0 (–)	9 (1.6)
Marital Status (N, %)***				
Married	80 (42.6)	84 (58.3)	181 (74.5)	345 (60.0)
Not Married	105 (55.8)	56 (38.9)	62 (25.5)	223 (38.8)
Missing	3 (1.6)	4 (2.8)	0 (–)	7 (1.2)
Income (N, %)***				
≤ $10,000 per year	71 (37.8)	10 (6.9)	35 (14.4)	116 (20.2)
$10,000–$50,000 per year	81 (43.1)	64 (44.4)	93 (38.2)	238 (41.4)
> $50,000 per year	10 (5.3)	22 (15.3)	66 (27.2)	98 (17.0)
Missing	26 (13.8)	48 (33.3)	49 (20.2)	123 (21.4)
Education (N, %)***				
Less than High School	38 (20.2)	14 (9.7)	21 (8.6)	73 (12.7)
High School/GED	97 (51.6)	48 (33.3)	65 (26.8)	210 (36.5)
Some College	31 (16.5)	47 (32.7)	48 (19.8)	126 (21.9)
College Grad/Post-Graduate	19 (10.1)	33 (22.9)	103 (42.4)	155 (27.0)
Other	2 (1.1)	2 (1.4)	5 (2.0)	9 (1.6)
Missing	1 (0.5)	0 (–)	1 (0.4)	2 (0.3)
Education (N, %)***				
Less than High School	38 (20.2)	14 (9.7)	21 (8.6)	73 (12.7)
High School/GED	97 (51.6)	48 (33.3)	65 (26.8)	210 (36.5)
Some College	31 (16.5)	47 (32.7)	48 (19.8)	126 (21.9)
College Grad/Post-Graduate	19 (10.1)	33 (22.9)	103 (42.4)	155 (27.0)
Other	2 (1.1)	2 (1.4)	5 (2.0)	9 (1.6)
Missing	1 (0.5)	0 (–)	1 (0.4)	2 (0.3)
OB Risk Score (N, %)				
0 risk conditions	145 (77.1)	102 (70.8)	206 (84.8)	453 (78.8)
1 risk condition	32 (17.0)	29 (20.1)	26 (10.7)	87 (15.1)
2 risk conditions	4 (2.2)	4 (2.8)	5 (2.0)	13 (2.3)
Missing	7 (3.7)	9 (6.3)	6 (2.5)	22 (3.8)
Diabetes (N, %)				
Yes	2 (1.1)	6 (4.1)	6 (2.5)	14 (2.4)
No	181 (96.3)	134 (93.1)	237 (97.5)	552 (96.0)
Missing	5 (2.6)	4 (2.8)	0 (–)	9 (1.6)
Smoking (N, %)***				
During Pregnancy	64 (34.1)	18 (12.5)	61 (25.1)	143 (24.9)
Not During Pregnancy	111 (59.0)	120 (83.4)	175 (72.0)	406 (70.6)
Missing	13 (6.9)	6 (4.1)	7 (2.9)	26 (4.5)
Infant/Birth Characteristics				
Birth type (N, %)				
Spontaneous/Induced/Non-Elective C-Section	173 (92.0)	128 (88.9)	218 (89.7)	519 (90.3)
Elective C-Section	15 (8.0)	16 (11.1)	23 (9.5)	54 (9.4)
Missing	0 (–)	0 (–)	2 (0.8)	2 (0.3)
Sex (N, %)				
Male	103 (54.8)	77 (53.5)	128 (52.7)	308 (53.6)
Female	85 (45.2)	67 (46.5)	115 (47.3)	267 (46.4)
Gestation Age at Birth (days)				
Average (SD)	271.4 (15.2)	271.6 (16.7)	274.2 (12.1)	272.6 (14.4)
Birth Weight (g)				
Average (SD)***	3151.5 (569.7)	3378.5 (617.8)	3407.1 (545.7)	3316.4 (582.8)

* α = 0.05.

** α = 0.01.

*** α = 0.001.

### Ethics Statement

All methods and procedures included in the current study were approved by the UC Irvine Institutional Review Board, the Cedars-Sinai Medical Center Institutional Review Board, and the Magee Women’s Hospital Institutional Review Board. All participants provided written informed consent. This consent procedure was approved by the institutional review boards of the participating institutions.

### Birth Weight, Gestational Age and Obstetric Risk Assessment

Information on birth weight was abstracted from the delivery medical record. For all participants, gestational age was determined by best obstetric estimate with a combination of last menstrual period and early uterine size, and was confirmed by obstetric ultrasonographic biometry using standard clinical criteria [30]. Obstetric risk was defined as the presence of major medical complications in the index pregnancy, i.e., vaginal bleeding, placenta abruptio, pregnancy-induced hypertension, preeclampsia, or infection. Risk conditions were ascertained by extensive medical chart review and coded as a binary variable (presence or absence of obstetric risk), as previously described [20]. Gestational diabetes was considered as a separate obstetric risk factor (and not part of the composite obstetric risk score) because, unlike the other obstetric risk conditions, it is associated with higher birth weight.

### Procedures

Participants attended multiple, serial study visits during pregnancy. Study assessments included structured psychosocial and medical interviews, questionnaires, fetal biometry ultrasound, and the collection of venous blood, saliva and urine. Maternal venous blood was collected in Paxgene blood DNA tubes and samples were stored at −80°C until DNA extraction.

### Resequencing and Genotyping

We resequenced the three key genes from the CRH pathway (CRH, CRH-BP, CRH-R1) in mouse-human hybrid somatic cell lines constructed from individuals representing three major American ethnic groups including 20 Africans, 19 Hispanics, and 20 Whites [31]. The hybrid cell lines were monosomic for human chromosome 8 (CRH), chromosome 5 (CRH-BP), and chromosome 17 (CRH-R1). The CRH and CRHBP genes were sequenced in their entirety. Due to its large size (51.6 kb), sequencing of CRHR1 was limited to functional regions, including the 5′ proximal promoter region, all exons, and partial intronic sequences adjacent to exons. We used genomic DNA from the hybrid cell lines for amplification of overlapping mid-sized fragments that covered CRH (2 fragments covering 4.4 kb), CRH-BP (12 fragments covering 18.8 kb), and CRHR1 (17 fragments covering 7.8 kb). These midsize fragments were sequenced using internal primers (300–500 bp intervals) with capillary-based methods (ABI DNA Analyzer 3730xl). Table S1A, B and C show general characteristics of the variants that were identified in the three genes by resequencing, including locations within gene regions, major/minor alleles, and minor allele frequencies for the two ethnic groups. Among these DNA variants identified by resequencing, we selected SNPs at these three loci for genotyping in lymphocyte DNA samples from 575 mother-offspring pairs using the ABI SNPlex platform. We excluded microsatellites, insertion/deletions, singleton SNPs (MAF<0.01), and SNPs that were not suitable for genotyping by SNPlex. After these exclusions, we used genotypic data for 58 SNPs for subsequent association analysis (9 SNPs for CRH, 30 SNPs for CRHBP, and 19 SNPs for CRH-R1). These SNPS are summarized in Figure S1 (CRH-BP), Figure S2 (CRH) and Figures S3 and S4 (CRH-R1).

### Statistical Analysis Approach and Methodology

SNP allele frequencies were estimated using the gene counting method and linkage disequilibrium between each pair of SNPs was defined by the formula presented by Weir [32] and estimated using an expectation-maximization (EM) algorithm. Tests of 1) goodness of fit of SNP genotype frequencies to the Hardy-Weinberg expectations, 2) differences in minor allele frequencies between races (and between the Southern California and Pennsylvania samples for each race, and 3) the differences in each of the discretely defined maternal and infant/birth characteristics between races were carried out using chi-square statistics. The F-test was employed to evaluate the statistical significance of the racial differences in the averages of the continuous variables. Multiple variable linear regression was employed to estimate the contribution of characteristics of the mother and child to predicting offspring birth weight in each race. A two stage statistical strategy was carried out to determine whether a gene region made a statistically significant contribution to explaining birth weight variation in all three races. Details are presented in Supporting Information S1.

## Results

A description of the mothers and infants included in our study is presented in Table 1. We completed studies of 575 mother-child pairs that were distributed among three racial/ethic groups. Fifty nine percent (340) of these pairs were recruited from Southern California and 41% (235) from Pittsburgh, Pennsylvania.

As depicted in Table 1, we observed statistically significant racial differences for maternal characteristics (except the prevalence of diabetes). Parity, sex of the child and the average gestational age at birth did not vary significantly among races. The average birth weight of infants of the Black mothers was 227 and 256 grams less than the offspring of Hispanic and White mothers, respectively.

Estimates of the contribution of variation in sex of child and characteristics of the mother in determining variation in birth weight are presented in Table 2 for two models. Model A estimates the average increase in birth weight for each day of gestational age and the effect of female sex of the child. Model B estimates the additional effects of one unit increase in BMI, multiparous status and smoking. We observed a statistically significant increase in birth weight per day of gestational age (25.9 to 26.8) and a statistically significant decrease in birth weight associated with female sex (−102.9 to −122.7) in each of the races when Model A was fitted to the data. There was no statistically significant evidence that Model A varied among races (F = 0.03, p = 0.99). Adding BMI, multiparous status and smoking in Model B significantly improved the prediction of birth weight in the samples of Black (p = 0.01) and White mothers (p = 0.006) but not in the sample of Hispanic mothers (p = 0.20). The size of the estimates of gestational age effects (26.2 to 26.8) and sex of the child (−88.3 to −112.4) were essentially unchanged in Model B compared to Model A, suggesting their effects are independent of BMI, multiparous status and smoking. Although estimates of the effects of BMI, multiparous status and smoking and their total contribution to explaining variation beyond gestational age and sex of the child varied among races, there was no statistical evidence that the ability of Model B to explain birth weight variation varied significantly among races (F = 0.45, p = 0.92).

**Table 2:** Risk factor effects on birth weight in grams.

	Blacks (N = 173)		Hispanics (N = 138)		Whites (N = 234)	
Trait	Model A^1^	Model B^2^	Model A	Model B	Model A	Model B
Gestation age/day	+26.0***	+26.2***	+25.9***	+26.8***	+26.8***	+26.6***
Female Child	−102.9*	−88.3	−122.7*	−110.9	−107.1*	−112.4**
BMI/(kg/m^2^)		+10.4**		+1.3		+4.1
Multiparous		+76.7		+169.7**		+98.7*
Smoked during pregnancy		−130.5**		−15.7		−200.5***

1 Model A: Birth weight = Gestation Age + Child Sex.

2 Model B: Birth weight = Gestation Age + Child Sex + Pre-pregnancy BMI + Parity + Smoking.

* Prob≤0.10.

** Prob≤0.05.

*** Prob≤0.01.

To provide a comprehensive survey of genetic variation in the three genes (CRH, CRH-BP, and CRH-R) we selected because they are involved in regulating the CRH pathway, we resequenced cell lines derived from 20 Blacks, 19 Hispanics, and 20 Whites representative of the three racial/ethnic groups included in our study. Tables S1, S2 and S3 present information on the identity, position, and relative allelic frequencies of the SNP variants characterized in these cell lines. Figures S1, S2, S3 and S4 give the relative frequencies of the minor allele for each of the SNPs identified by the resequencing that were selected for genotyping in the 173 Black, 138 Hispanic and 234 White mothers who participated in our study.

The results of the analyses to evaluate the contribution of SNP variation to explaining birth weight variation beyond the traditional risk factors are presented in Tables 3 and 4 for each of the three gene regions. Each of the three gene regions made a significant contribution to explaining birth weight variation in Blacks. Two gene regions (CRH-BP and CRH-R1) made a significant contribution to variation in Hispanics and one region (CRH-BP) made a significant contribution in Whites (Table 3). The contribution of the genotypes determined by the selected SNPs to explaining birth weight variation ranged from 3.45% and 1.55% for models A and B, respectively, for White infants to 12.39% and 6.11% for models A and B, respectively, for Black infants. SNP variation in the CRH-BP gene region explained 6.11%, 9.53% and 1.55% of birth weight variation adjusted for the complete set of risk factors in Blacks, Hispanics and Whites, respectively. The SNPs that were selected to represent the contribution of each gene region to explaining variation in birth weight beyond that explained by either Model A or Model B for each race are listed in Table 4.

**Table 3:** Significant SNP genotype effects on birth weight beyond risk factors.

		Blacks (N = 173)			Hispanics (N = 138)			Whites (N = 234)		
Gene Region	Model	Number of Tests	Significant N	% Genetic Variance^3^	Number of Tests	Significant N	% Genetic Variance^3^	Number of Tests	Significant N	% Genetic Variance^3^
*CRHBP*	A^1^	29	6	12.39**	30	9	11.85**	25	2	3.45**
	B^2^	29	4	6.11**	30	9	9.53**	25	1	1.55**
*CRH*	A^1^	9	2	3.70**	8	0	0.00	6	0	0.00
	B^2^	9	1	1.84**	8	0	0.00	6	0	0.00
*CRHR1*	A^1^	19	2	5.06***	17	1	2.67**	19	0	0.00
	B^2^	19	2	5.27***	17	1	2.73**	19	0	0.00

1 Model A: Birth weight = Gestation Age + Child Sex + SNP.

2 Model B: Birth weight = Gestation Age + Child Sex + Pre-pregnancy BMI + Parity + Smoking + SNP.

3 % Genetic Variance from combined genotypes.

* Prob≤0.10.

** Prob≤0.05.

*** Prob≤0.01.

**Table 4:** SNPs with significant effects beyond risk factors on birth weight.

	Blacks (N = 173)		Hispanics (N = 138)		Whites (N = 234)	
Gene Region	Model A^1^	Model B^2^	Model A	Model B	Model A	Model B
*CRHBP*			rs41272246	rs41272246	rs41272246	
	rs32897					
						76255599*
	rs7718461	rs7718461	rs7718461	rs7718461		
	rs7728378	rs7728378	rs7728378	rs7728378		
	rs75319082					
			rs7721519	rs7721519		
	rs10055255	rs10055255	rs10055255	rs10055255		
			rs1875999	rs1875999		
	rs1053989	rs1053989	rs1053989	rs1053989	rs1053989	
			rs2135078	rs2135078		
			rs2174444	rs2174444		
*CRH*	rs12721510					
	rs28364018	rs28364018				
*CRHR1*	rs12936511	rs12936511				
	rs28364028	rs28364028	rs28364028	rs28364028		

1 Model A: Birth weight = Gestation Age + Child Sex + SNP.

2 Model B: Birth weight = Gestation Age + Child Sex + Pre-pregnancy BMI + Parity + Smoking + SNP.

* GRCh37.2 position (no rs number assigned for this SNP).

On the basis of the results of the analyses presented in Tables 3 and 4 we next turned to identifying a subset of SNPs that represented the CRH-BP gene region in all three races. Six SNPs were selected to mark genetic variation in Blacks, nine were selected in Hispanics, and three in Whites (Table 4). Three SNPs each marked significant variation in Blacks and Hispanics, and one SNP (rs1053989) marked significant variation in all three races. The location of these SNPs is presented in Figure S1A. A search for the tag SNPs that represent variation in the CRH-BP gene region identified rs1053989 in each of the races.

**Figure 1:**
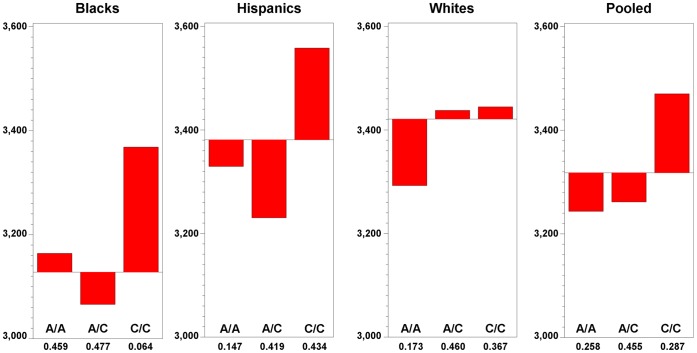
Birth weight means in grams for CRH-BP SNP rs1053989 genotypes. Birth weights adjusted by length of gestation, child sex, maternal BMI, parity, smoking.

Table 5 presents the estimates of the genotype effects of SNP rs1053989 for Model A and Model B. The pattern of the size of the genotype effects and their statistical significance are essentially the same for models A and B, i.e., they are independent of whether BMI and parity and smoking are in the model. The estimates of genotype effects when Model B is considered that are presented in Table 5 are plotted in Figure 1, where the relative frequencies of the three genotypes are included.

**Table 5:** Estimates of effects of mother’s risk factors and mother’s genotype (CRHBP SNP rs1053989) on infant birth weight in grams.

Risk Factors Model A				
	Blacks (N = 172)	Hispanics (N = 136)	Whites (N = 226)	Pooled(N = 534)
Race (Black)	–	–	–	−133***
Race (Hispanic)	–	–	–	+37
GA (per day)	+27***	+27***	+27***	+27***
Sex (female)	−94	−99	−103*	−101***
*CRHBP* rs1053989				
A/A	−239*	−239**	−210***	−184***
A/C	−330***	−353***	−30	−186***
C/C	Reference	Reference	Reference	Reference
**Risk Factors Model B**				
	**Blacks (N = 172)**	**Hispanics (N = 136)**	**Whites (N = 226)**	**Pooled (N = 534)**
Race (Black)	–	–	–	−159***
Race (Hispanic)	–	–	–	−4
GA (per day)	+27***	+27***	+27***	+27***
Sex (female)	−78	−85	−110**	−98***
BMI (per unit)	+9**	−2	+4	+5*
Parity (multiparous)	+86	+135*	+92	+92**
Smoking (yes)	−141**	−12	−199***	−145***
*CRHBP* rs1053989				
A/A	−215*	−241**	−154*	−155***
A/C	−312**	−341***	−8	−168***
C/C	Reference	Reference	Reference	Reference

* Prob≤0.10.

** Prob≤0.05.

*** Prob≤0.01.

The A allele explains a large and statistically significant dominant effect on the decrease in birth weight in Blacks (A/A = 3173, A/C = 3075, C/C = 3378) and Hispanics (A/A = 3323, A/C = 3224, C/C = 3552) and a recessive effect in Whites (A/A = 3291, A/C = 3436, C/C = 3442). Those Black and Hispanic mothers carrying the A allele had infants whose birth weight was on average 254 and 302 grams, respectively, less than infants having C/C mothers. Those White mothers homozygous for the A allele had infants who were on average 148 grams less than those infants having A/C and C/C mothers. The relative frequency of the A allele in Black mothers was significantly greater than in Hispanic and White mothers (Figure 1). Black mothers carried the A allele (relative frequency = 0.703) approximately twice as often as the Hispanic (relative frequency = 0.356) and White mothers (relative frequency = 0.402).

## Discussion

In our study of the relation between birth weight and sequence variation in the CRH family of genes, we found that variation in the 3′ untranslated region (UTR) of the CRH-BP gene, marked by SNP rs1053989, explained a significant amount of variation in birth weight in Whites, Hispanics and Blacks. The influence of sequence variation in the CRH-BP gene on birth weight in our study is similar in magnitude to the reported influence of cigarette smoking [33] and about half that of maternal cocaine use [34]. In our view, in contrast to the pathologic nature of the exposures of smoking and cocaine, common variation in the CRH-BP gene is more likely to represent a physiologic contribution to birth weight. This speculation deserves directed evaluation in prospective fashion, with particular attention to the postnatal implications of birth weight as a relevant phenotype.

With respect to previous explorations of the genetic contribution to birth weight, Freathy et al conducted a meta-analysis of six genome-wide association studies of newborn genotype and identified two loci (rs900400 near *LKR1* and *CCNL1*, and rs9883204 in *ADCY5*)[35]. These investigators estimated that newborns carrying four “birth weight-lowering” alleles were 113 grams smaller at birth than newborns carrying zero or one of these alleles. Our present investigation identified sequence variation with a contribution to birth weight at least as large or larger than the four allele model identified by Freathy et al. Further, our candidate gene approach with a targeted survey of a physiological pathway influencing fetal growth identified sequence variation in the CRH-BP gene not identified in the GWAS meta-analysis approach. Of note, our findings are in maternal genotype while those of Freathy et al are in the newborn genome. The genome-wide association approach and the candidate gene approach, in this instance, produce complementary findings that together permit insights into birth weight provided by neither approach alone. Our finding of a maternal gene variant influencing newborn birth weight is an example of an indirect maternal genetic effect [36]. These effects are important in understanding the development of an individual’s phenotype as well as understanding the evolution of the genetic architecture responsible for that phenotype.

The effect was observed across the three racial/ethnic groups, suggesting that this genetic effect, while variable in magnitude, is persistent across subpopulations that vary with respect to ancestry and environment. Confidence in a gene-phenotype association is enhanced when that association is present across groups of individuals that vary with respect to geography, ancestry, and environment.

The effect of CRH-BP sequence variation on birth weight is unchanged after adjusting for maternal BMI, parity, and smoking, suggesting that this variation represents an independent contribution to fetal growth beyond those clinical and epidemiological factors commonly used in attempts to explain variation in birth weight [29]. While these, and other, environmental factors may exert an influence on fetal growth, and, thus, birth weight, our adjusted analyses suggest that our identified genetic effect is not explained by unmeasured confounding from these factors.

The biological mechanisms linking CRH-BP to fetal growth and birth weight are unknown. In human pregnancy, maternal CRH is related to offspring birth weight and fat mass [37]. We speculate that CRH-BP may regulate bioavailability of CRH and thus influence fetal growth. The molecular function of rs1053989 has been described in the literature. SNP rs1053989 alters a putative binding site for microRNA miR33a in the 3′ UTR of CRH-BP (exon 7) (CAAAGCAACGTGCAATA **C/A** AA)[38]. Interestingly, rs1053989 disrupts the miR33a “seed” sequence (CAATA **C/A** A) that must be perfectly complementary for binding of microRNAs with recognition sites in 3′ UTRs of targeted genes. This evolutionarily conserved microRNA is encoded within intron 16 of the gene for sterol-responsive element-binding protein 2 transcription factor (SREBP2 on chromosome 22) that regulates cholesterol homeostasis. Previous functional studies have shown that miR33a regulates expression of genes in cellular pathways of cholesterol transport, including ABCA1 (ATP binding cassette transporter) and the lysosomal transporter protein NPC1 (Niemann-Pick disease, type C1) [39]–[40]. This microRNA is co-expressed with SREBP2 in many different tissues including placenta, indicating that miR33a may be a global regulator of cholesterol transport and metabolism [39]. Maternal cholesterol is associated with fetal growth; maternal levels of HDL cholesterol are inversely related to infant birth weight [41]. ABCA1 is present in human placenta and is localized largely at the basolateral and to some extent at the apical side of first trimester villous cytotrophoblast cell membranes [42]. Placental expression of ABCA1 is thought to play an important role in maternal-fetal cholesterol transfer. A recent study that assessed the expression profile of miRNAs and their predicted target genes in placentas from preeclampsia and preterm labor (both of which are associated with decreased birth weight) reported altered expression of a number or miRNAs associated several target genes including CRH and CRH-BP [43].

This finding raises the opportunity for future study, and, possibly, patient care advances. Our data do not permit insight into the relative contribution of CRH-BP sequence variation to the various components of newborn weight such as skeletal, lean and fat mass. Further study of the body composition of newborns and the influence of genotype will serve to further dissect biological mechanism of influence. The contribution of sequence variation in the CRH-BP gene to physiologic and pathologic variation in the growth of the human fetus ought to be assessed prospectively in a population of women with rigorously ascertained environmental contributors to birth weight. Our finding also raises the possibility that sequence variation in CRH-BP might influence the clinical ability of assessment of fetal growth to distinguish the fetus with pathologic growth from the fetus with physiological growth restriction/impairment. Observational and randomized trials are warranted to evaluate the clinical utility of birth weight customization models that include genotype to distinguish newborns at risk for adverse outcome from those not at risk for adverse outcome. Current strategies show promise in this regard but are limited by their inability to explain sufficient physiological variation in birth weight. This is particularly an issue in attempts to identify the small-for-gestational-age and large-for-gestational-age newborns at risk for adverse outcome [4], [44]. The incorporation of genotype into antenatal customization models may add precision and clinical utility to these tools for assessing the adequacy of fetal growth.

## Supporting Information

Figure S1 **Map of the Corticotrophin Releasing Hormone Binding Protein (CRH-BP) Gene – 5g11.2–13.3.**
(TIF)

Figure S2 **Map of the Corticotrophin Releasing Hormone (CRH) Gene – 8q13.**
(TIF)

Figure S3 **Map of the Corticotrophin Releasing Hormone Receptor 1 (CRH-R1) 17q12–q22 (1 of 2).**
(TIF)

Figure S4 **Map of the Corticotrophin Releasing Hormone Receptor 1 (CRH-R1) 17q12–q22 (2 of 2).**
(TIF)

Table S1 CRH-BP Gene.
(DOC)

Table S2 CRH Gene.
(DOC)

Table S3 CRH-R1 Gene.
(DOC)

Supporting Information S1 **Statistical Methods.**
(DOC)
