# Supplementary figures and images for: Variation in the Maternal Corticotrophin Releasing Hormone-Binding Protein (CRH-BP) Gene and Birth Weight in Blacks, Hispanics and Whites
Source: PLoS One. 2012 Sep 11;7(9):e43931. doi: 10.1371/journal.pone.0043931 (PMC3439482; doi:10.1371/journal.pone.0043931)

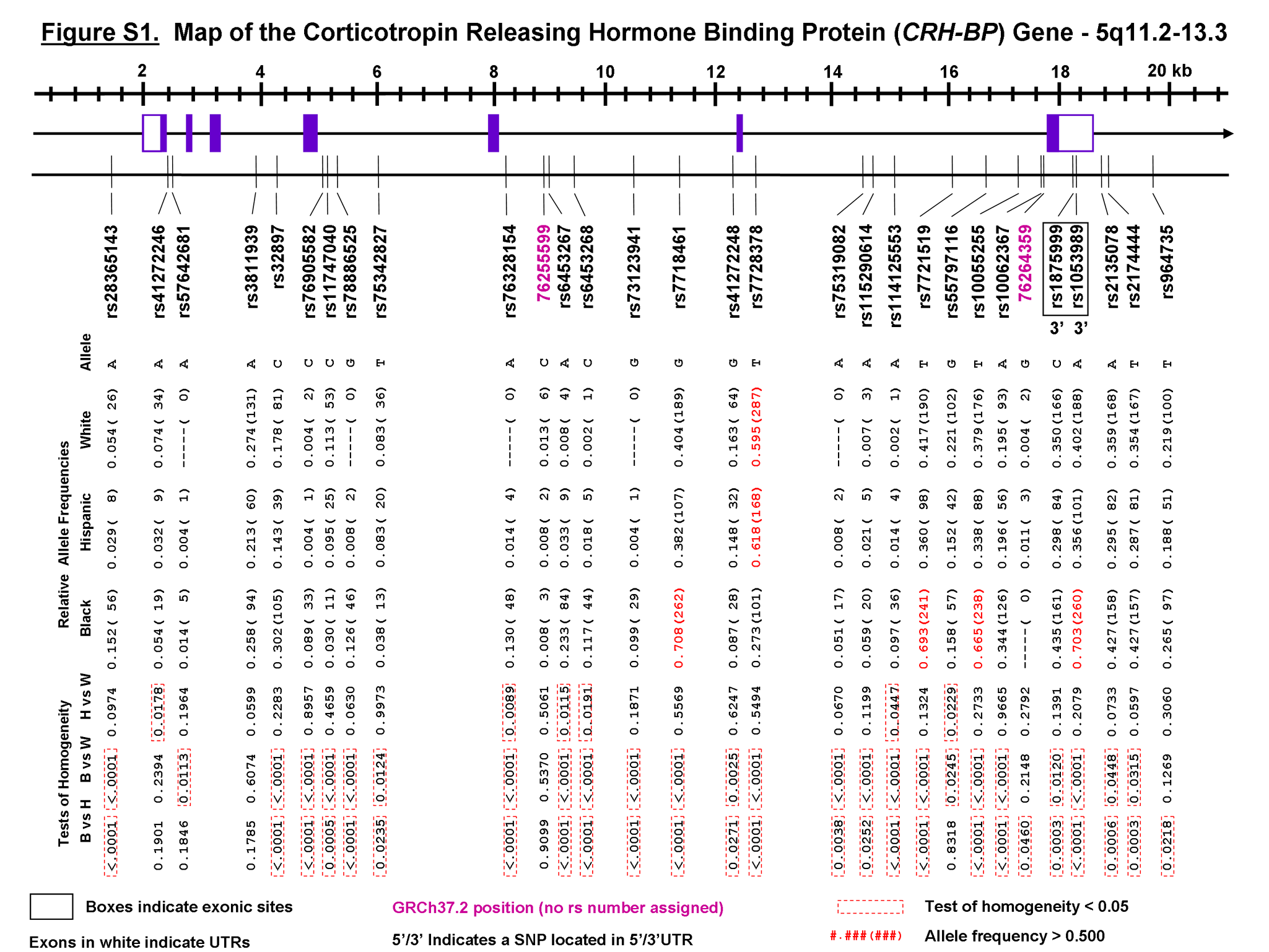

Supplement: Figure S1 — Map of the Corticotrophin Releasing Hormone Binding Protein (CRH-BP) Gene – 5g11.2–13.3. (TIF) [file pone.0043931.s001.tif]

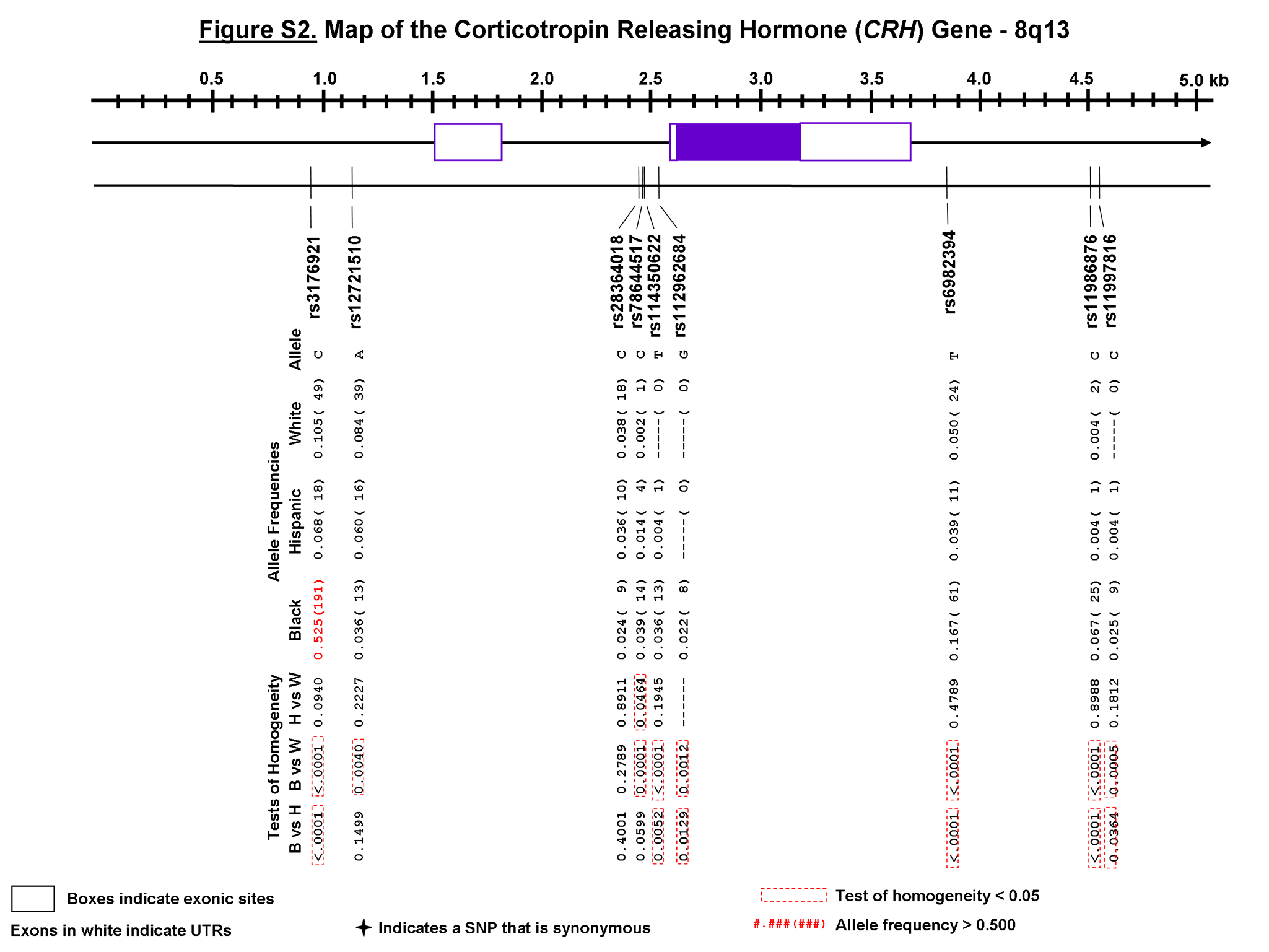

Supplement: Figure S2 — Map of the Corticotrophin Releasing Hormone (CRH) Gene – 8q13. (TIF) [file pone.0043931.s002.tif]

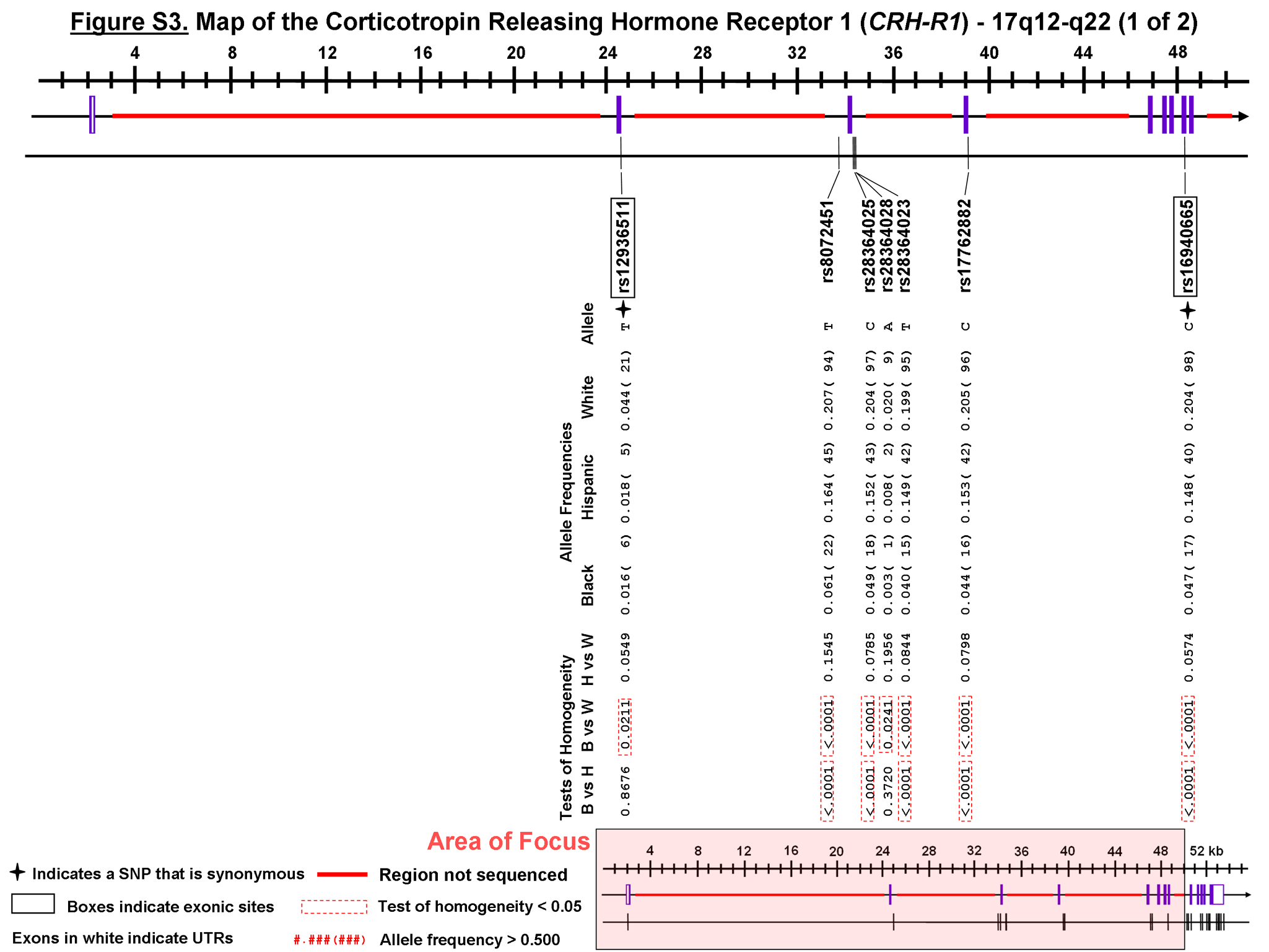

Supplement: Figure S3 — Map of the Corticotrophin Releasing Hormone Receptor 1 (CRH-R1) 17q12–q22 (1 of 2). (TIF) [file pone.0043931.s003.tif]

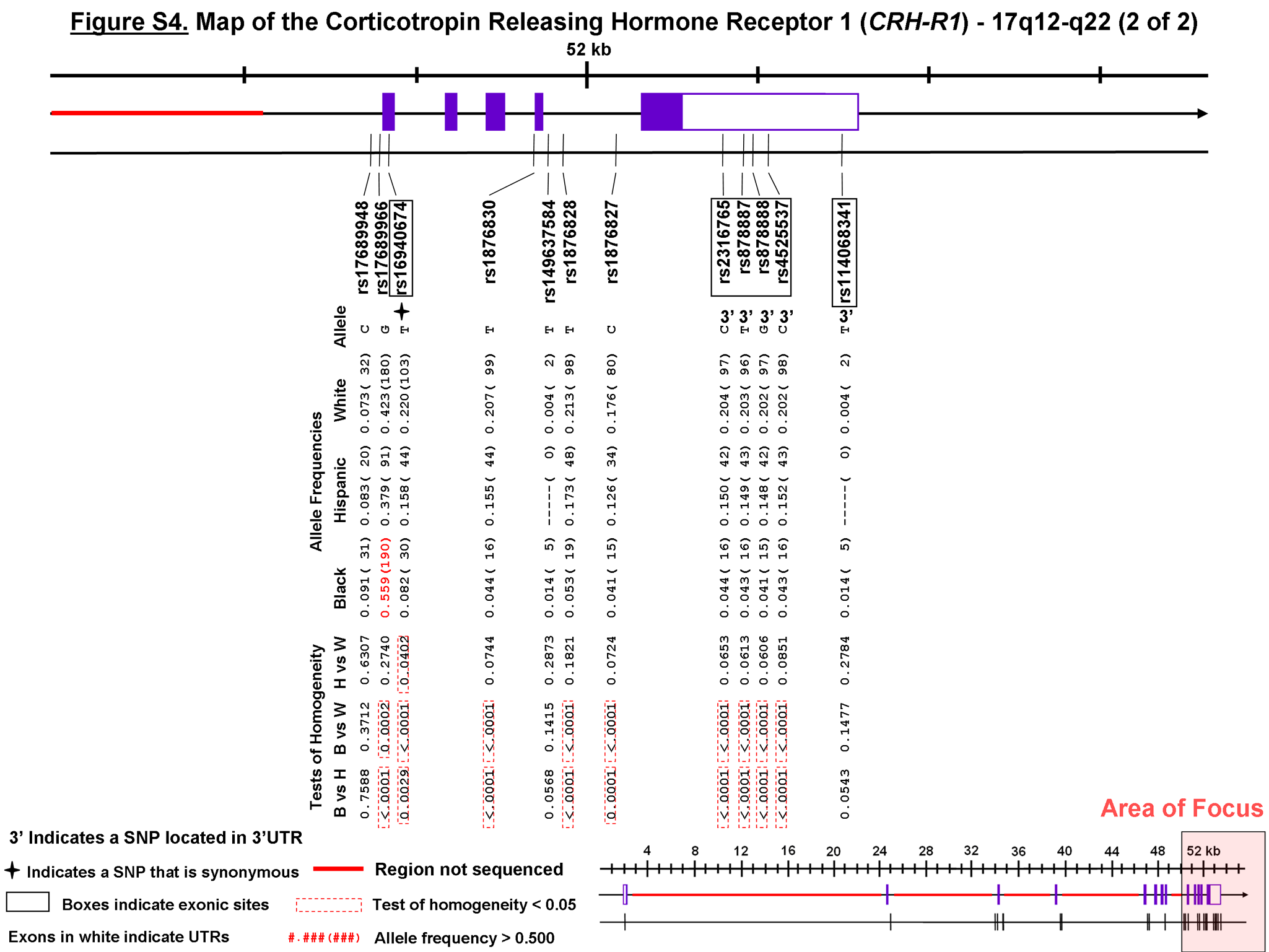

Supplement: Figure S4 — Map of the Corticotrophin Releasing Hormone Receptor 1 (CRH-R1) 17q12–q22 (2 of 2). (TIF) [file pone.0043931.s004.tif]
