# Supplementary material for: Variation in the Maternal Corticotrophin Releasing Hormone-Binding Protein (CRH-BP) Gene and Birth Weight in Blacks, Hispanics and Whites
Source: PLoS One. 2012 Sep 11;7(9):e43931. doi: 10.1371/journal.pone.0043931 (PMC3439482; doi:10.1371/journal.pone.0043931)
Supplement: Table S1 — CRH-BP Gene. (DOC) [file pone.0043931.s005.doc]

**Supporting Information Table S1 – *CRH-BP*** Gene

|  |  | **GRCh37.2** |  | **Gene** | **Gene** |  |  | **Minor Allele Frequencies** | | |
| --- | --- | --- | --- | --- | --- | --- | --- | --- | --- | --- |
| **Position** | **rs number** | **Position** |  | **Structure** | **Region** | **aa subn** | **Major > Minor** | **Blacks** | **Hispanics** | **Whites** |
| -874 | rs28365143 | 76248086 | *1 | 5' flank |  |  | G>A |  |  |  |
| -322 |  | 76248638 |  | promoter |  |  | G>C | 0.027 | 0 | 0 |
| 103 | rs41272246 | 76249062 | * | intron1 |  |  | C>A | 0.139 | 0 | 0 |
| 174 | rs57642681 | 76249133 | * | intron1 |  |  | C>A | 0.111 | 0 | 0 |
| 544 | rs78115247 | 76249503 |  | exon2 | coding | Pro | G>A | 0 | 0 | 0.027 |
| 577 | rs1715771 | 76249536 |  | intron2 |  |  | C>G | 0.059 | 0.273 | 0.568 |
| 751 |  | 76249710 |  | intron2 |  |  | A>G | 0 | 0.15 | 0 |
| 983 | rs34347676 | 76249942 |  | exon3 | coding | Glu | G>A | 0.056 | 0 | 0 |
| 986 | rs34761141 | 76249945 |  | exon3 | coding | Pro | C>G | 0.083 | 0 | 0 |
| 1180 |  | 76250139 |  | intron2 |  |  | T>G | 0.056 | 0 | 0 |
| 1368 | rs11951796 | 76250327 |  | intron3 |  |  | C>T | 0.028 | 0 | 0 |
| 1378 | rs35292061 | 76250337 |  | intron3 |  |  | ->+ | 0.389 | 0.394 | 0.368 |
| 1419 | rs7721799 | 76250378 |  | intron3 |  |  | G>A | 0.028 | 0.194 | 0.081 |
| 1438 | rs11741842 | 76250397 |  | intron3 |  |  | C>T | 0 | 0.032 | 0.135 |
| 1628 | rs3811939 | 76250587 | * | intron3 |  |  | G>A | 0.275 | 0.395 | 0.231 |
| 2013 | rs32897 | 76250972 | * | intron3 |  |  | T>C | 0.556 | 0.139 | 0.179 |
| 2768 | rs76905582 | 76251727 | * | intron4 |  |  | G>C | 0.05 | 0 | 0 |
| 2885 | rs11747040 | 76251844 | * | intron4 |  |  | T>C | 0.025 | 0.027 | 0.125 |
| 3072 | rs78886525 | 76252031 | * | intron4 |  |  | A>G | 0.05 | 0 | 0 |
| 3329 | rs114642073 | 76252288 |  | intron4 |  |  | C>T | 0.05 | 0 | 0 |
| 3339 |  | 76252298 |  | intron4 |  |  | C>T | 0 | 0.027 | 0 |
| 3376 |  | 76252335 |  | intron4 |  |  | C>T | 0.025 | 0 | 0 |
| 3416 | rs32898 | 76252375 |  | intron4 |  |  | G>A | 0.05 | 0.351 | 0.425 |
| 3529 | rs113106833 | 76252488 |  | intron4 |  |  | G>T | 0.25 | 0.351 | 0.175 |
| 3727 | rs75342827 | 76252686 | * | intron4 |  |  | C>T | 0.103 | 0.053 | 0.1 |
| 4275 | rs111744183 | 76253234 |  | intron4 |  |  | C>T | 0.182 | 0.032 | 0 |
| 5132 |  | 76254091 |  | intron4 |  |  | T>A | 0.027 | 0.027 | 0 |
| 5941 | rs76328154 | 76254900 | * | intron5 |  |  | G>A | 0.179 | 0.026 | 0 |
| 6517 |  | 76255476 |  | intron5 |  |  | +>- | 0.025 | 0 | 0 |
| 6640 |  | 76255599 | * | intron5 |  |  | G>C | 0.025 | 0 | 0.025 |
| 6796 | rs151057785 | 76255755 |  | intron5 |  |  | C>T | 0.05 | 0 | 0 |
| 6797 | rs6453267 | 76255756 | * | intron5 |  |  | G>A | 0.2 | 0.026 | 0 |
| 7010 | rs75876015 | 76255969 |  | intron5 |  |  | G>C | 0.028 | 0 | 0 |
| 7203 | rs6453268 | 76256162 | * | intron5 |  |  | G>C | 0.225 | 0.029 | 0 |
| 7631 |  | 76256590 |  | intron5 |  |  | G>T | 0.026 | 0 | 0 |
| 7649 | rs35214445 | 76256608 |  | intron5 |  |  | ->+ | 0.359 | 0.25 | 0.222 |
| 8204 | rs73123941 | 76257163 | * | intron5 |  |  | A>G | 0.15 | 0 | 0 |
| 8606 | rs79101755 | 76257565 |  | intron5 |  |  | T>C | 0.026 | 0 | 0 |
| 8623 | rs116235782 | 76257582 |  | intron5 |  |  | T>C | 0.051 | 0 | 0 |
| 8808 | rs12521643 | 76257767 |  | intron5 |  |  | C>T | 0.077 | 0.162 | 0.175 |
| 8809 |  | 76257768 |  | intron5 |  |  | G>A | 0.026 | 0 | 0 |
| 8818 |  | 76257777 |  | intron5 |  |  | C>T | 0.067 | 0.027 | 0 |
| 8930 | rs112250835 | 76257889 |  | intron5 |  |  | A>T | 0.026 | 0 | 0 |
| 9089 | rs7718461 | 76258048 | * | intron5 |  |  | A>G | 0.676 | 0.459 | 0.421 |
| 9091 |  | 76258050 |  | intron5 |  |  | +>- | 0.027 | 0 | 0 |
| 9093 |  | 76258052 |  | intron5 |  |  | A>G | 0.027 | 0 | 0 |
| 9145 |  | 76258104 |  | intron5 |  |  | T>G | 0 | 0 | 0.026 |
| 9449 | rs78726046 | 76258408 |  | intron5 |  |  | G>A | 0.097 | 0 | 0 |
| 9459 | rs60668317 | 76258418 |  | intron5 |  |  | A>G | 0.065 | 0.167 | 0.171 |

1 * denotes SNPs genotyped in the samples of mothers studied

**Supporting Information Table S1 – *CRH-BP*** Gene

|  |  | **GRCh37.2** |  | **Gene** | **Gene** |  |  | **Minor Allele Frequencies** | | |
| --- | --- | --- | --- | --- | --- | --- | --- | --- | --- | --- |
| **Position** | **rs number** | **Position** |  | **Structure** | **Region** | **aa subn** | **Major > Minor** | **Blacks** | **Hispanics** | **Whites** |
| 9740 | rs3841124 | 76258699 |  | intron5 |  |  | +>- | 0.425 | 0.27 | 0.25 |
| 9752 |  | 76258711 |  | intron5 |  |  | G>T | 0 | 0 | 0.15 |
| 9965 |  | 76258924 |  | intron5 |  |  | +>- | 0.028 | 0 | 0 |
| 10052 | rs41272248 | 76259011 | * | intron5 |  |  | A>G | 0.056 | 0.135 | 0.179 |
| 10155 | rs78284172 | 76259114 |  | intron5 |  |  | T>G | 0.029 | 0 | 0 |
| 10158 | rs147534880 | 76259117 |  | intron5 |  |  | A>G | 0 | 0.054 | 0 |
| 10391 | rs7728378 | 76259350 | * | intron6 |  |  | C>T | 0.231 | 0.514 | 0.564 |
| 10857 | rs57038272 | 76259816 |  | intron6 |  |  | C>T | 0.154 | 0.211 | 0.175 |
| 11023 |  | 76259982 |  | intron6 |  |  | ->+ | 0.051 | 0 | 0 |
| 11160 |  | 76260119 |  | intron6 |  |  | C>T | 0.026 | 0 | 0 |
| 11161 |  | 76260120 |  | intron6 |  |  | ->+ | 0.026 | 0 | 0 |
| 11162 |  | 76260121 |  | intron6 |  |  | G>A | 0.026 | 0 | 0 |
| 11164 |  | 76260123 |  | intron6 |  |  | ->+ | 0.026 | 0 | 0 |
| 11538 | rs112988924 | 76260497 |  | intron6 |  |  | G>A | 0.077 | 0 | 0 |
| 11646 |  | 76260605 |  | intron6 |  |  | C>T | 0.026 | 0 | 0 |
| 11903 |  | 76260862 |  | intron6 |  |  | ->+ | 0.069 | 0 | 0 |
| 12005 |  | 76260964 |  | intron6 |  |  | G>T | 0.032 | 0 | 0 |
| 12281 | rs75319082 | 76261240 | * | intron6 |  |  | G>A |  |  |  |
| 12440 | rs115290614 | 76261399 | * | intron6 |  |  | G>A | 0.075 | 0 | 0 |
| 12782 |  | 76261741 |  | intron6 |  |  | G>A | 0 | 0 | 0.026 |
| 12839 | rs114125553 | 76261798 | * | intron6 |  |  | G>A | 0.225 | 0.026 | 0 |
| 12973 | rs143189959 | 76261932 |  | intron6 |  |  | C>A | 0.125 | 0 | 0 |
| 13586 | rs149783174 | 76262545 |  | intron6 |  |  | C>A | 0.025 | 0 | 0 |
| 13805 | rs7721519 | 76262764 | * | intron6 |  |  | C>T | 0.725 | 0.474 | 0.425 |
| 13813 | rs114512211 | 76262772 |  | intron6 |  |  | T>C | 0.025 | 0 | 0 |
| 14397 |  | 76263356 |  | intron6 |  |  | +>- | 0 | 0.026 | 0 |
| 14443 | rs55797116 | 76263402 | * | intron6 |  |  | A>G | 0.15 | 0.211 | 0.179 |
| 14476 |  | 76263435 |  | intron6 |  |  | +>- | 0.05 | 0 | 0 |
| 14546 | rs111926350 | 76263505 |  | intron6 |  |  | T>C | 0.025 | 0.026 | 0 |
| 14588 | rs146492566 | 76263547 |  | intron6 |  |  | G>A | 0.083 | 0 | 0 |
| 14853 | rs142223880 | 76263812 |  | intron6 |  |  | G>A | 0.026 | 0 | 0 |
| 15030 |  | 76263989 |  | intron6 |  |  | +>- | 0.026 | 0 | 0 |
| 15034 | rs10055255 | 76263993 | * | intron6 |  |  | T>A | 0.263 | 0.514 | 0.564 |
| 15395 | rs10062367 | 76264354 | * | intron6 |  |  | G>A | 0.25 | 0.237 | 0.25 |
| 15400 |  | 76264359 | * | intron6 |  |  | A>G | 0.05 | 0.026 | 0 |
| 15537 |  | 76264496 |  | intron6 |  |  | G>T | 0.025 | 0 | 0 |
| 15903 |  | 76264862 |  | exon7 | 3'utr |  | +>- | 0.075 | 0 | 0 |
| 15946 | rs147081585 | 76264905 |  | exon7 | 3'utr |  | C>G | 0.05 | 0 | 0 |
| 16023 | rs1875999 | 76264982 | * | exon7 | 3'utr |  | T>C | 0.575 | 0.432 | 0.325 |
| 16076 | rs1053989 | 76265035 | * | exon7 | 3'utr |  | A>C | 0.275 | 0.514 | 0.575 |
| 16286 | rs80156591 | 76265245 |  | exon7 | 3'utr |  | T>C | 0 | 0.028 | 0 |
| 16509 | rs2135078 | 76265468 | * | 3'flank |  |  | G>A | 0.575 | 0.421 | 0.325 |
| 16563 | rs2174444 | 76265522 | * | 3'flank |  |  | C>T | 0.575 | 0.421 | 0.325 |
| 16873 |  | 76265832 |  | 3'flank |  |  | A>G | 0.1 | 0.184 | 0.15 |
| 17381 | rs33989123 | 76266340 |  | 3'flank |  |  | ->+ | 0.5 | 0.432 | 0.312 |
| 17387 |  | 76266346 |  | 3'flank |  |  | A>C | 0.103 | 0.189 | 0.156 |
| 17410 | rs964734 | 76266369 |  | 3'flank |  |  | G>T | 0.55 | 0.432 | 0.344 |
| 17420 | rs964735 | 76266379 | * | 3'flank |  |  | C>T | 0.375 | 0.243 | 0.188 |
| 17488 | rs10514082 | 76266447 |  | 3'flank |  |  | A>G | 0.1 | 0.189 | 0.156 |

1 * denotes SNPs genotyped in the samples of mothers studied
