# Supplementary material for: Variation in the Maternal Corticotrophin Releasing Hormone-Binding Protein (CRH-BP) Gene and Birth Weight in Blacks, Hispanics and Whites
Source: PLoS One. 2012 Sep 11;7(9):e43931. doi: 10.1371/journal.pone.0043931 (PMC3439482; doi:10.1371/journal.pone.0043931)
Supplement: Table S2 — CRH Gene. (DOC) [file pone.0043931.s006.doc]

**Supporting Information Table S2 – *CRH*** Gene

|  |  | **GRCh37.2** |  | **Gene** | **Gene** |  |  | **Minor Allele Frequencies** | | |
| --- | --- | --- | --- | --- | --- | --- | --- | --- | --- | --- |
| **Position** | **rs number** | **Position** |  | **Structure** | **Region** | **aa subn** | **Major > Minor** | **Blacks** | **Hispanics** | **Whites** |
| -1809 |  | 67091521 |  | 5'flank |  |  | A>G | 0.026 | 0 | 0 |
| -1706 | rs116492734 | 67091418 |  | 5'flank |  |  | T>G | 0.105 | 0 | 0 |
| -1667 | rs3176921 | 67091379 | *1 | 5'flank |  |  | T>C | 0.737 | 0.194 | 0.125 |
| -1470 | rs12721510 | 67091182 | * | promotor |  |  | C>A |  |  |  |
| -630 |  | 67090342 |  | intron1 |  |  | G>T | 0.026 | 0 | 0 |
| -618 | rs28364014 | 67090330 |  | intron1 |  |  | G>C | 0.105 | 0 | 0 |
| -617 | rs78420283 | 67090329 |  | intron1 |  |  | T>C | 0.026 | 0 | 0 |
| -598 | rs73693934 | 67090310 |  | intron1 |  |  | G>T | 0.079 | 0.167 | 0 |
| -596 | rs73693933 | 67090308 |  | intron1 |  |  | T>G | 0.079 | 0.167 | 0 |
| -338 |  | 67090050 |  | intron1 |  |  | A>T | 0 | 0 | 0.025 |
| -278 | rs76978322 | 67089990 |  | intron1 |  |  | G>A | 0.026 | 0 | 0 |
| -158 | rs28364018 | 67089870 | * | intron1 |  |  | A>C | 0.105 | 0.083 | 0.025 |
| -136 | rs78644517 | 67089848 | * | intron1 |  |  | A>C | 0.026 | 0 | 0 |
| -130 | rs114350622 | 67089842 | * | intron1 |  |  | A>T |  |  |  |
| -73 | rs112962684 | 67089785 | * | intron1 |  |  | A>G | 0.026 | 0 | 0 |
| 124 | rs72556398 | 67089662 |  | exon2 | coding | Leu | G>C | 0.105 | 0 | 0 |
| 145 |  | 67089641 |  | exon2 | coding | Ala | G>A | 0.026 | 0 | 0 |
| 361 | rs6159 | 67089425 |  | exon2 | coding | Gly | A>C | 0.778 | 0.333 | 0.179 |
| 967 |  | 67088819 |  | exon2 | 3'utr |  | +>- | 0.026 | 0 | 0 |
| 1309 | rs6982394 | 67088477 | * | 3'flank |  |  | G>T |  |  |  |
| 1381 |  | 67088405 |  | 3'flank |  |  | A>C | 0 | 0 | 0.025 |
| 1964 | rs11986876 | 67087822 | * | 3'flank |  |  | T>C | 0.081 | 0.167 | 0 |
| 2022 | rs11997816 | 67087764 | * | 3'flank |  |  | A>C | 0.027 | 0 | 0 |

1 * denotes SNPs genotyped in the samples of mothers studied
