# Supplementary material for: Variation in the Maternal Corticotrophin Releasing Hormone-Binding Protein (CRH-BP) Gene and Birth Weight in Blacks, Hispanics and Whites
Source: PLoS One. 2012 Sep 11;7(9):e43931. doi: 10.1371/journal.pone.0043931 (PMC3439482; doi:10.1371/journal.pone.0043931)
Supplement: Table S3 — CRH-R1 Gene. (DOC) [file pone.0043931.s007.doc]

**Supporting Information Table S3 – *CRH-R1*** Gene

|  |  | **GRCh37.2** |  | **Gene** | **Gene** |  |  | **Minor Allele Frequencies** | | |
| --- | --- | --- | --- | --- | --- | --- | --- | --- | --- | --- |
| **Position** | **rs number** | **Position** |  | **Structure** | **Region** | **aa subn** | **Major > Minor** | **Blacks** | **Hispanics** | **Whites** |
| -794 | rs62057073 | 43861117 |  | 5'flank |  |  | C>T | 0.029 | 0.179 | 0.257 |
| -449 |  | 43861462 |  | promoter |  |  | G>C | 0 | 0 | 0.059 |
| -388 |  | 43861523 |  | promoter |  |  | +>- | 0 | 0 | 0.029 |
| -58 | rs28364020 | 43861853 |  | exon1 | 5'utr |  | C>T | 0.212 | 0.29 | 0.156 |
| -27 | rs116702137 | 43861884 |  | exon1 | 5'utr |  | C>G | 0.061 | 0 | 0 |
| 22490 | rs12936511 | 43884402 | *1 | exon2 | coding | Pro | C>T | 0.057 | 0.061 | 0 |
| 31804 | rs8072451 | 43893716 | * | intron2 |  |  | C>T | 0.056 | 0.176 | 0.222 |
| 31821 |  | 43893733 |  | intron2 |  |  | G>C | 0.139 | 0.088 | 0.111 |
| 31839 | rs8073146 | 43893751 |  | intron2 |  |  | A>G | 0.056 | 0.176 | 0.222 |
| 31859 | rs10585044 | 43893771 |  | intron2 |  |  | +>- | 0.056 | 0.176 | 0.194 |
| 31861 | rs66475418 | 43893773 |  | intron2 |  |  | +>- | 0.056 | 0.176 | 0.194 |
| 31972 | rs8077279 | 43893884 |  | exon3 | coding | Pro | T>C | 0.028 | 0 | 0 |
| 32190 | rs28364025 | 43894102 | * | intron3 |  |  | T>C | 0.029 | 0.194 | 0.226 |
| 32199 | rs28364028 | 43894111 | * | intron3 |  |  | T>A | 0 | 0 | 0.033 |
| 32247 | rs28364023 | 43894159 | * | intron3 |  |  | C>T | 0.029 | 0.207 | 0.233 |
| 36669 |  | 43898581 |  | intron3 |  |  | C>T | 0 | 0.031 | 0 |
| 36975 | rs17762882 | 43898887 | * | intron4 |  |  | T>C | 0.031 | 0.167 | 0.207 |
| 37051 | rs17689653 | 43898963 |  | intron4 |  |  | A>T | 0.031 | 0.167 | 0.207 |
| 44814 | rs17425752 | 43906726 |  | intron5 |  |  | A>C | 0 | 0.182 | 0.200 |
| 44916 | rs17689882 | 43906828 |  | intron5 |  |  | G>A | 0 | 0.182 | 0.200 |
| 45061 | rs75638861 | 43906973 |  | intron5 |  |  | G>A | 0 | 0 | 0.033 |
| 45727 | rs16940662 | 43907639 |  | intron6 |  |  | C>A | 0.057 | 0.121 | 0.143 |
| 45730 | rs3029044 | 43907642 |  | intron6 |  |  | ->+ | 0.029 | 0.152 | 0.257 |
| 45833 | rs1876831 | 43907745 |  | intron6 |  |  | C>T | 0.029 | 0.152 | 0.222 |
| 45840 | rs114469083 | 43907752 |  | intron6 |  |  | T>A | 0.057 | 0 | 0 |
| 45984 | rs16940665 | 43907896 | * | exon7 | coding | Thr | T>C | 0.029 | 0.120 | 0.250 |
| 46054 | rs16940668 | 43907966 |  | intron7 |  |  | G>A | 0.029 | 0.120 | 0.258 |
| 46239 | rs16940671 | 43908151 |  | intron7 |  |  | C>T | 0 | 0.172 | 0.250 |
| 46240 | rs16940672 | 43908152 |  | intron7 |  |  | C>T | 0 | 0.172 | 0.250 |
| 46565 | rs56396707 | 43908477 |  | intron8 |  |  | +>- | 0.028 | 0.188 | 0.229 |
| 48490 | rs17689948 | 43910402 | * | intron8 |  |  | G>C | 0.029 | 0.107 | 0.125 |
| 48543 | rs17689966 | 43910455 | * | intron8 |  |  | G>A | 0.429 | 0.536 | 0.531 |
| 48595 | rs16940674 | 43910507 | * | exon9 | coding | Cys | C>T | 0.029 | 0.167 | 0.222 |
| 48675 |  | 43910587 |  | intron9 |  |  | G>A | 0.057 | 0 | 0 |
| 48726 | rs115157945 | 43910638 |  | intron9 |  |  | G>A | 0.029 | 0 | 0 |
| 48741 | rs242950 | 43910653 |  | intron9 |  |  | C>T | 0.257 | 0.100 | 0.111 |
| 49056 | rs34969299 | 43910968 |  | intron10 |  |  | G>A | 0.206 | 0 | 0 |
| 49119 |  | 43911031 |  | intron10 |  |  | G>A | 0.029 | 0 | 0 |
| 49124 | rs16940676 | 43911036 |  | intron10 |  |  | G>A | 0.029 | 0.176 | 0.237 |
| 49440 | rs1876830 | 43911352 | * | intron11 |  |  | C>T | 0.029 | 0.176 | 0.222 |
| 49512 | rs41457044 | 43911424 |  | intron12 |  |  | C>T | 0.029 | 0.176 | 0.222 |
| 49523 | rs149637584 | 43911435 | * | intron12 |  |  | C>T | 0.029 | 0 | 0 |
| 49531 | rs1876829 | 43911443 |  | intron12 |  |  | T>C | 0.029 | 0.176 | 0.222 |
| 49613 | rs1876828 | 43911525 | * | intron12 |  |  | C>T | 0.029 | 0.176 | 0.222 |
| 49920 | rs1876827 | 43911832 | * | intron12 |  |  | T>C | 0 | 0.154 | 0.241 |
| 49986 | rs16940677 | 43911898 |  | intron12 |  |  | C>T | 0 | 0.143 | 0.233 |
| 50247 | rs16940681 | 43912159 |  | exon13 | 3'utr |  | G>C | 0.028 | 0.156 | 0.243 |
| 50363 | rs937 | 43912275 |  | exon13 | 3'utr |  | ->+ | 0.028 | 0.182 | 0.216 |
| 50370 | rs28364021 | 43912282 |  | exon13 | 3'utr |  | C>T | 0.028 | 0.182 | 0.237 |

1 * denotes SNPs genotyped in the samples of mothers studied

**Supporting Information Table S3 – *CRH-R1*** Gene

|  |  | **GRCh37.2** |  | **Gene** | **Gene** |  |  | **Minor Allele Frequencies** | | |
| --- | --- | --- | --- | --- | --- | --- | --- | --- | --- | --- |
| **Position** | **rs number** | **Position** |  | **Structure** | **Region** | **aa subn** | **Major > Minor** | **Blacks** | **Hispanics** | **Whites** |
| 50382 | rs28364026 | 43912294 |  | exon13 | 3'utr |  | G>A | 0.028 | 0.091 | 0.053 |
| 50430 | rs28364032 | 43912342 |  | exon13 | 3'utr |  | G>A | 0.028 | 0.121 | 0.158 |
| 50502 |  | 43912414 |  | exon13 | 3'utr |  | G>C | 0 | 0.030 | 0 |
| 50542 | rs2316765 | 43912454 | * | exon13 | 3'utr |  | T>C | 0.028 | 0.182 | 0.237 |
| 50578 | rs878886 | 43912490 |  | exon13 | 3'utr |  | C>G | 0.028 | 0.182 | 0.243 |
| 50670 | rs878887 | 43912582 | * | exon13 | 3'utr |  | C>T | 0.028 | 0.182 | 0.257 |
| 50723 | rs878888 | 43912635 | * | exon13 | 3'utr |  | A>G | 0.029 | 0.188 | 0.235 |
| 50811 | rs4525537 | 43912723 | * | exon13 | 3'utr |  | T>C | 0.029 | 0.182 | 0.235 |
| 50874 | rs4640231 | 43912786 |  | exon13 | 3'utr |  | G>C | 0 | 0.118 | 0.235 |
| 50893 | rs28364027 | 43912805 |  | exon13 | 3'utr |  | A>G | 0.056 | 0.059 | 0 |
| 50918 | rs4482334 | 43912830 |  | exon13 | 3'utr |  | T>C | 0.028 | 0.176 | 0.229 |
| 51118 | rs16940686 | 43913030 |  | exon13 | 3'utr |  | G>T | 0.059 | 0.071 | 0 |
| 51196 |  | 43913108 |  | exon13 | 3'utr |  | G>A | 0.029 | 0 | 0 |
| 51242 | rs114068341 | 43913154 | * | exon13 | 3'utr |  | C>T | 0.059 | 0 | 0 |
| 51309 |  | 43913221 |  | 3'flank |  |  | G>A | 0 | 0 | 0.029 |
|  |  |  |  |  |  |  |  |  |  |  |

1 * denotes SNPs genotyped in the samples of mothers studied
