# Supplementary material for: Variation in the Maternal Corticotrophin Releasing Hormone-Binding Protein (CRH-BP) Gene and Birth Weight in Blacks, Hispanics and Whites
Source: PLoS One. 2012 Sep 11;7(9):e43931. doi: 10.1371/journal.pone.0043931 (PMC3439482; doi:10.1371/journal.pone.0043931)
Supplement: Supporting Information S1 — Statistical Methods. (DOC) [file pone.0043931.s008.doc]

Supporting Information S1: Statistical Methods

A two stage statistical strategy was carried out to determine whether a gene region made a statistically significant contribution to explaining birth weight variation in all three races. In the first step, in each race stratum we identified a subset of SNPs for each of the three gene regions that individually made a statistically significant contribution to explaining variability in birth weight. In the second step we tested the statistical significance of the contribution of the multi-SNP genotypes determined by the selected SNPs to birth weight variation in each race stratum. Finally, we carried out an analysis of linkage disequilibrium to identify tag SNPs of the subset of SNPs that each made a statistical significant contribution in each race stratum (1). Established risk factors, including an obstetric risk score mentioned previously, were considered in a backward selection but the regression factors listed below were the only ones that stayed in the model for birth weight.

To test the effects of each SNP we compared two linear effects models using a likelihood ratio chi-square test. First, the sample was balanced on all variables included in a full (“complete”) model. This model included a set of risk factors for low birth weight and the SNP. The “reduced” model included the risk factors only. Two complete models were considered. In the first, gestational age and sex of the child were included with the SNP. In the second, gestational age, sex of the child, parity and mother’s BMI and smoking status during pregnancy were included with the SNP. Gestational age and BMI were entered into these models as continuous variables, and sex of the child, parity, smoking and SNP genotypes were treated as categorical variables. Each of the SNPs was represented by three genotypes. The most common homozygote was used as the reference genotype group. Comparing the -2 log likelihoods of a complete and a reduced model yields a two-degree of freedom chi-square test of SNP genotype effects. At this step we used a nominal α level of 0.05 to determine the statistical significance of the contribution of each SNP to explaining birth weight variability.

The race-independent contribution of a gene region was based on statistically significant evidence that the multi-SNP genotype variation determined by the selected SNPs made a significant contribution to birth weight in all three races. To estimate the contribution of the multi-SNP genotypes determined by the SNPs that were significant in each of the three gene regions considered in this study, we used the method introduced by Boerwinkle and Sing (1986)(2). This method provides a bias-corrected estimate of the proportion of phenotypic variance attributable to the gene region marked by the genotypes defined by the statistically significant SNPs. The estimate is defined as,

$$\frac{\sum_{j=1}^{J} \frac{n_{j}}{n}(\hat{\mu}_{j}-\hat{\mu}_{.})^{2}-\left(\frac{J-1}{n}\right)\hat{\sigma}^{2}}{\sum_{j=1}^{J} \frac{n_{j}}{n}(\hat{\mu}_{j}-\hat{\mu}_{.})^{2}+\hat{\sigma}^{2}\left[1-\left(\frac{J-1}{n}\right)\right]}$$

where *n_j_* is the number of individuals in the *j*th genotype class, $\hat{\mu}_{j}=\frac{\sum_{i=1}^{n_{j}} \hat{y}_{i}}{n_{j}}$is the predicted mean for the *j*th genotype class (estimated by a complete model that includes the risk factors and the multi-SNP genotypes), $\hat{\mu}_{.}=\frac{\sum_{j=1}^{J} \sum_{i=1}^{n_{j}} \hat{y}_{ij}}{n}$is the overall predicted mean, and $\hat{\sigma}^{2}$ is the estimated model variance. This formulation was applied to estimate the contribution of SNP variation to variation in birth weight adjusted for the contribution of child and mother’s characteristics discussed above.

1. Carlson CS, Eberle MA, Rieder MJ, Yi Q, Kruglyak L, Nickerson DA. Selecting a maximally informative set of single-nucleotide polymorphisms for association analyses using linkage disequilibrium. Am J Hum Genet. 2004 Jan;74(1):106-20.

2. Boerwinkle E, Sing CF. Bias of the contribution of single-locus effects to the variance of a quantitative trait. Am J Hum Genet. 1986 Jul;39(1):137-44.
